# Supplementary material for: The interactome of intact mitochondria by cross-linking mass spectrometry provides evidence for coexisting respiratory supercomplexes
Source: Mol Cell Proteomics. 2017 Dec 8;17(2):216–32. doi: 10.1074/mcp.RA117.000470 (PMC5795388; doi:10.1074/mcp.RA117.000470)
Supplement: Supplemental Data [file supp_RA117.000470_133922_0_supp_23978_fzffwf.zip › spectra_annotation/mito_spectra_annotation/264-1-10-1-9-1.pdf]

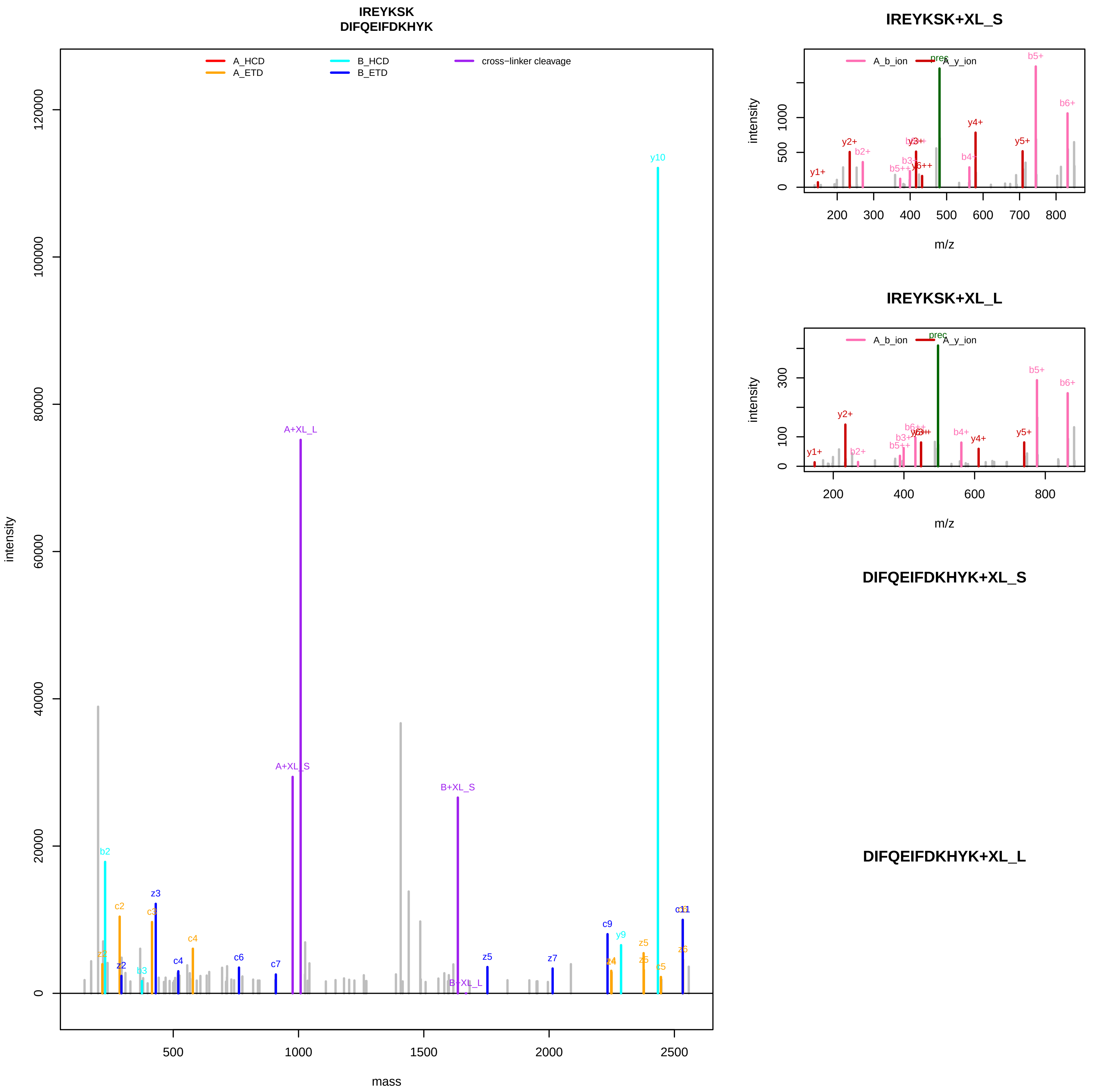
IREYKSK
DIFQEIFDKHYK
A_HCD
A_ETD
B_HCD
B_ETD
cross−linker cleavage
intensity
mass
y10
A+XL_L
A+XL_S
B+XL_S
B+XL_L
b2
c2
c3
z3
c4
c6
c7
z5
z7
c9
y9
z5
c11
IREYKSK+XL_S
IREYKSK+XL_L
DIFQEIFDKHYK+XL_S
DIFQEIFDKHYK+XL_L
A_b_ion
A_y_ion
prec
m/z
y1+
y2+
b2+
y3+
y4+
y5+
b4+
b5+
b6+
b5++
b3+
y6++
b6++
